# Disruptions of Host Immunity and Inflammation by *Giardia Duodenalis:* Potential Consequences for Co-Infections in the Gastro-Intestinal Tract

**DOI:** 10.3390/pathogens4040764

**Published:** 2015-11-10

**Authors:** James A. Cotton, Christina B. Amat, Andre G. Buret

**Affiliations:** 1 Cumming School of Medicine, University of Calgary, Calgary, AB T2N 4N1, Canada; E-Mail: jacotton@ucalgary.ca; 2 Department of Biological Sciences, University of Calgary, Calgary, AB T2N 1N4, Canada; E-Mail: cb.amat@ucalgary.ca; 3 Inflammation Research Network, University of Calgary, Calgary, AB T2N 1N4, Canada; 4 Host-Parasite Interactions, University of Calgary, Calgary, AB T2N 1N4, Canada

**Keywords:** *Giardia duodenalis*, host-parasite interactions, diarrheal disease, inflammation, immunomodulation

## Abstract

*Giardia duodenalis* (syn. *G. intestinalis*, or *G. lamblia*) is a leading cause of waterborne diarrheal disease that infects hundreds of millions of people annually. Research on *Giardia* has greatly expanded within the last few years, and our understanding of the pathophysiology and immunology on this parasite is ever increasing. At peak infection, *Giardia* trophozoites induce pathophysiological responses that culminate in the development of diarrheal disease. However, human data has suggested that the intestinal mucosa of *Giardia*-infected individuals is devoid of signs of overt intestinal inflammation, an observation that is reproduced in animal models. Thus, our understanding of host inflammatory responses to the parasite remain incompletely understood and human studies and experimental data have produced conflicting results. It is now also apparent that certain *Giardia* infections contain mechanisms capable of modulating their host’s immune responses. As the oral route of *Giardia* infection is shared with many other gastrointestinal (GI) pathogens, co-infections may often occur, especially in places with poor sanitation and/or improper treatment of drinking water. Moreover, *Giardia* infections may modulate host immune responses and have been found to protect against the development of diarrheal disease in developing countries. The following review summarizes our current understanding of the immunomodulatory mechanisms of *Giardia* infections and their consequences for the host, and highlights areas for future research. Potential implications of these immunomodulatory effects during GI co-infection are also discussed.

## 1. Introduction

Infections caused by the protozoan parasite *Giardia duodenalis* (syn. *G. intestinalis*, *G. lamblia*) are a major cause of waterborne diarrheal disease worldwide, and are estimated to cause ~280 million infections annually [1]. However, the advent of molecular assays with higher sensitivity for *Giardia* trophozoites and cysts suggests this number may be underestimated [2,3]. This parasite has long been overlooked for its ability to cause diarrheal disease, and it was not until the 20th century that it was definitively identified as a causative agent of infectious diarrheal disease [4]. *Giardia* is now included on the WHO’s Neglected Disease Initiative [5], and research on *Giardia* appears to be increasing [6]. At the height of infection, *Giardia* trophozoites induce pathophysiological processes that result in a malabsorptive diarrheal disease (reviewed in [7]). Symptoms classically associated with Giardiasis include diarrhea, abdominal pain, nausea, vomiting, and anorexia. However, infected individuals can also develop extra-intestinal and post-infectious complications [8,9]. Chronic extraintestinal sequelae may affect the joints, the skin, the eyes, and even the central nervous system, and the mechanisms are unknown [8,9]. For reasons that remain obscure, *Giardia* infections cause a spectrum of symptoms ranging from asymptomatic carriage through to chronic diarrheal disease [10]. Although chronic *Giardia* infection tends to occur in immunocompromised individuals, it has been reported in patients without obvious immunodeficiency (reviewed in [10]). In addition, asymptomatic infection has been observed in developed countries following re-infection with the same isolate [11]. *Giardia* is currently subdivided into eight distinct genetic assemblages labelled as assemblage “A” through “H” [12,13]. Humans are susceptible to infection from assemblage “A” and “B” isolates. Some studies have suggested that symptom development may in part be assemblage-dependent, but results are largely inconclusive [14,15,16,17,18]. However, *in vivo* studies in mice have demonstrated differences in the pathogenicity of assemblage A and B isolates [19], and genomic analysis of assemblage A and B isolates indicates substantial disparity between the two groups [20,21]. This has led to the proposition that assemblage “A” and “B” *Giardia* isolates are actually unique *Giardia* species, a topic of ongoing debate in the scientific literature [20,22].

Our understanding of the pathophysiology and immunity in giardiasis is drastically improving, yet discrepancies in study results persist and much remains to be learned [23,24,25,26]. Several parasites are known to affect various aspects of their host’s pro-inflammatory responses [27,28], and recent findings indicate that *Giardia* actively modulates host inflammatory responses (as referenced below). This is particularly important when considering that this parasite is often found in association with a variety of pro-inflammatory gastrointestinal (GI) pathogens. The purpose of this review is to summarize our current understanding surrounding the immunomodulatory mechanisms of *Giardia* and discuss potential consequences of this phenomenon during GI co-infection.

## 2. Does *Giardia duodenalis* Induce Pro-Inflammatory Responses?

The GI mucosal barrier is comprised of two main components: a secreted mucus layer and the intestinal epithelium. This structure restricts luminal contents, including various exogenous and endogenous antigens, from contacting underlying host tissues and, subsequently, inducing GI pro-inflammatory responses (reviewed in [29,30]). Dysfunction of the GI mucosal barrier is observed in chronic GI inflammatory states, such as Crohn’s disease, and contributes to disease progression [31,32]. Moreover, a broad variety of GI pathogens induce GI barrier dysfunction during infection [33,34]. It has been well established that *Giardia* infections cause intestinal barrier dysfunction via a variety of mechanisms, including activation of myosin light chain kinase and increased rates of intestinal epithelial apoptosis [35,36,37,38,39]. At the height of infection, parasite numbers exceed 10^6^ trophozoites per centimetre of gut; coupled with increases in intestinal permeability, it is possible that the presence of copious amounts of exogenous parasitic material could induce pro-inflammatory intestinal responses via translation to subepithelial spaces. In addition, it has recently been suggested that certain *G. duodenalis* isolates may be capable of invading into host tissues in *in vivo* gerbil models [40]. Despite this, evidence supporting the development of any overt inflammatory response is lacking, and human studies and experimental research have produced conflicting results. Histological analysis of *Giardia*-infected individuals and *in vivo* *Giardia muris* infections shows small intestinal mucosal tissues are devoid of signs of significant inflammation [41,42,43,44]. Small increases in intra-epithelial lymphocyte numbers and mast cell hyperplasia post-infection have been observed [45]. Microarray analysis of jejunal tissues from assemblage E-infected cattle demonstrated downregulation of genes associated with inflammatory and immune responses, as well as immune cell migration; this was associated with increased expression of the anti-inflammatory transcription factor peroxisome proliferation activation receptor γ (PPARγ) [46]. Moreover, co-incubation *in vitro* of intestinal epithelial monolayers and assemblage A *Giardia* trophozoites does not produce pro-inflammatory cytokines and/or chemokines [47,48]. Rather, microarray analysis from studies *in vitro* demonstrated that assemblage A *G. duodenalis* induces a chemokine profile that is different from the host responses commonly seen with other GI pathogens, whereby parasites significantly increased mRNA levels of CCL2, CCL20, and CXCL1-3 [49]. In children populations from developing countries, *Giardia* infections also have been found to reduce the incidence of diarrheal disease and fever, and decrease serum C-reactive protein (CRP) levels, which is a common marker of inflammation [50]. Together, the data available to date would indicate that *Giardia* infections fail to induce, and perhaps downregulate, factors associated with intestinal inflammatory responses within its hosts.

However, findings from some human studies and experimental evidence suggest that *Giardia* infections may induce pro-inflammatory intestinal responses. Subsets of human patients with chronic assemblage B infection were shown to develop microscopic duodenal inflammation and displayed elevated fecal calprotectin levels [51]. Experimental infections *in vivo* with *G. duodenalis* assemblage B have been associated with post-infectious neutrophil (polymorphonuclear leukocyte, PMN) infiltration in the terminal ileum [52] or even more robust intestinal inflammatory responses [53]. Excretory/secretory products from these assemblage B trophozoites activated pro-inflammatory mitogen activated protein kinase (MAPK) and nuclear factor of κB (NF-κB) signaling pathways in intestinal epithelial monolayers and produced pro-inflammatory cytokines and chemokines, including tumor necrosis factor α (TNF-α) and the potent PMN chemoattractant interleukin-8 (CXCL8) [54]. In addition, reports examining human patients [55,56] and ruminants [57] suggested that at least some *Giardia* infections may cause eosinophilia; this was also shown in *in vivo* infections whereby *Giardia* assemblage B H3 infections [58] or parasite excretory/secretory products [59] caused intestinal recruitment of eosinophils. It is interesting that *in vivo* and *in vitro* experimental data suggest assemblage B isolates induce intestinal pro-inflammatory responses, while similar observations have not been demonstrated for assemblage A isolates, or with *Giardia muris* infections *in vivo*. Some reports correlate assemblage B infections with more severe symptomatology, while others have suggested the same for assemblage A infections [14,15,16,17]. More research is needed to determine whether assemblage B infections may indeed induce intestinal inflammatory responses and more severe diarrheal disease. The differences between the pro-inflammatory capabilities of assemblage A and B *Giardia* isolates warrant further investigation.

## 3. Polymicrobial GI Infections Involving *Giardia*

The above observations that *Giardia* infections may or may not induce pro-inflammatory intestinal responses also need to be considered in the context of GI co-infections. *Giardia* infections occur upon ingestion of contaminated food or water, or directly via the fecal-oral route (reviewed in [60]). This method of acquisition is common to various GI pathogens, and, therefore, can easily result in polymicrobial GI infections in the appropriate setting. Due to poor hygiene and lack of appropriate water treatment facilities, polymicrobial infections are more frequently observed in developing countries but can also occur within developed countries [61,62]. Infections are more likely to cause symptomatic infection in individuals living in developed countries compared to those living in developing countries [63,64]. Indeed, *Giardia* infections have been reported concurrently with pro-inflammatory pathogens such as *Helicobater pylori*, *Vibrio cholera*, enteropathogenic *Escherichia coli*, norovirus, and rotavirus [65,66,67,68], and others have found *Giardia* infections in association with *Ascaris* sp., *Cryptosporidium* sp., *Clostridium difficile*, and *Salmonella* sp. [69,70,71]. However, our knowledge of polymicrobial GI infections and intestinal inflammatory responses involving *Giardia* is poorly understood, and, to date, much evidence is anecdotal. Several human studies suggest that *Giardia* infections may protect against the development of diarrheal disease and, potentially, the development of intestinal inflammatory responses [72]. Tanzanian children infected with *Giardia* were shown to have reduced incidence of diarrheal disease and fever, and lower serum C-reactive protein levels [50]. However, this study did not look for the presence of polymicrobial GI infections. *Giardia* infections in humans have also been associated with other GI pathogens or identified in control patients not experiencing diarrhea [67]. *Giardia* infections may reduce the severity of symptoms associated with rotavirus infection [68], yet separate studies have suggested that *Giardia* infections enhanced the severity of rotavirus infection [73]. To date, we have no scientific explanation for these observations. Apart from a single *in vivo* study showing acute intestinal infection with *Trichinella spiralis* increases host susceptibility to *Giardia* GS/M infection [74], *in vivo* polymicrobial infections involving *Giardia* parasites have not been examined.

## 4. *Giardia* and Immunomodulation

Accumulating experimental evidence suggests that *Giardia* infections are also capable of modulating pro-inflammatory responses to other stimuli via several mechanisms. Observations that *Giardia* infections can protect against the development of diarrheal disease are consistent with the immunomodulatory capabilities of the parasite. Indeed, acute GI inflammatory responses represent a collection of cellular and humoral effector responses and involve a variety of different cell types and mediators; several of these have been shown to contribute to the development of diarrheal disease. For example, infection with enterohaemorrhagic *E. coli* causes chloride hypersecretion, a major driving force for diarrheal disease, via mechanisms that require PMN infiltration [75]. Research has demonstrated that certain *Giardia* infections are capable of attenuating recruitment of pro-inflammatory leukocytes and decreasing nitric oxide (NO) production (as referenced below). In addition, evidence is accumulating that *Giardia* infections may modulate other pro-inflammatory events. However, these mechanisms have not been fully characterized. The following sections will describe the immunomodulatory mechanisms of *Giardia* and describe how this may result in the attenuation of diarrheal disease during GI co-infection.

### 4.1. Giardia and the Intestinal Mucus Layer

The entire GI tract is lined with a layer of mucus of varying thickness with a structural backbone comprised of mucin glycoproteins dissolved in luminal water. In the colon, this layer can be further subdivided into two separate layers: a dense, inner mucus layer largely devoid of bacterial populations and an outer, loosely packed outer layer containing various bacterial populations [76,77]. In the intestinal tract, the primary mucus constituent is the mucin-2 (MUC2) protein [76,77,78]. Preliminary research in our lab has demonstrated *in vivo Giardia* assemblage B GS/M isolate infections in mice damages the small intestinal mucus layer by degrading the MUC2 protein and inducing the hypersecretion of mucus in the small intestine and colon, resultantly leading to mucin depletion from goblet cells; this culminated in a weakened mucus layer and facilitated disease (unpublished data). Furthermore, studies monitoring mucus disruption during *in vivo Giardia* GS/M infections have observed an increase in bacterial translocation across the epithelial barrier, but this was not associated with an increase in pro-inflammatory markers at the point of acute infection [52]. Separate *in vivo* studies have demonstrated that pro-inflammatory enteropathogens, such as *H. pylori*, *Entamoeba histolytica,* and *Trichuris muris*, alter the mucus layer and this contributes to the initiation or exacerbation of GI disease [79,80,81,82]. Similarly, modulation or aberrant assembly of the mucus layer is often associated with intestinal inflammation and increased expression of pro-inflammatory cytokines including interleukin (IL)-1β, IL-4, IL-6, CXCL8 IL-13, and TNF-α [83,84,85,86]. Finally, *in vivo* studies using mice devoid of Muc2 have revealed that the mucus layer plays an important role in protection against GI infection from pro-inflammatory enteropathogens, such as *E. histolytica* and *T. muris*, and deletion of this gene results in exacerbated intestinal inflammatory responses [87,88,89]. Similarly, disruption or aberrant expression of MUC2 has been observed in patients with chronic intestinal inflammatory disorders, such as ulcerative colitis [90,91,92]. Collectively, these results demonstrate that disruption of the intestinal mucus layer is largely associated with GI inflammation. It remains to be determined why disruption of the mucus layer during *Giardia* infections fails to elicit pro-inflammatory intestinal responses. Moreover, it remains to be seen how *Giardia* co-infections may alter host pro-inflammatory responses and/or alter susceptibility to co-infecting GI pathogens.

### 4.2. Giardia and Neutrophil Recruitment

The tissue accumulation of polymorphonuclear leukocytes or neutrophils (PMNs) is a hallmark of numerous bacterial, viral, and parasitic GI infections. PMNs are myeloid-derived innate immune cells essential to host defence against a variety of bacterial and fungal pathogens, and they possess various anti-microbial mechanisms, including the ability to phagocytose infectious agents, secrete anti-microbial proteases, and release neutrophil extracellular traps (NETs) (reviewed in [93]). In the absence of pro-inflammatory stimuli, PMNs are kept in a non-activated state within the bone marrow and circulation. During an acute inflammatory response, increased expression and production of PMN chemoattractants promotes PMN activation and recruitment into tissues, including the GI tract (reviewed in [94,95]). Certain PMN chemoattractants are capable of inducing the transepithelial migration of PMNs; this process occurs following PMN contact with the basolateral surface of the intestinal epithelium and results in functional changes to both PMNs and intestinal epithelial cells (IECs) [96]. Importantly, PMN infiltration can induce pathophysiological responses that result in water and solute loss and, hence, diarrheal disease, and *in vivo* and *in vitro* experiments have suggested this may involve PMN-mediated intestinal barrier dysfunction and/or anion secretion [75,97,98,99]. Collectively, these results demonstrate the importance PMNs have in contributing to diarrheal disease.

Recent studies have shown that *Giardia* infections may attenuate intestinal PMN recruitment. Notably, these observations have been recorded with assemblage A, the genotype that has been postulated not to induce overt intestinal pro-inflammatory responses (see above). For example, *Giardia* assemblage A decreased granulocyte infiltration and cytokines and chemokines involved in PMN recruitment after intra-rectal instillation of pro-inflammatory *Clostridium difficile* toxin A/B; these effects were not observed with *in vivo Giardia* assemblage B GS/M infections [100]. This study was also the first to demonstrate that co-incubation of *Giardia* trophozoites with inflamed colonic mucosal biopsy tissues from patients with active Crohn’s disease decreased supernatant levels of numerous pro-inflammatory mediators, including those involved in PMN recruitment [100]. Further studies went on to identify potential immunomodulatory molecules involved in this process. The findings demonstrated that assemblage A *Giardia* cathepsin B (catB) cysteine proteases degraded CXCL8 induced by pro-inflammatory interleukin-1β, or by *Salmonella enterica* serovar Typhimurium, and attenuated CXCL8-induced PMN chemotaxis; these effects were not observed with assemblage B GS/M trophozoites at early time points and, potentially, occur via different mechanisms [101]. These studies highlight a hitherto unidentified anti-inflammatory capability for *Giardia* infections and, more specifically, *Giardia* catB proteases. Another recent study shows that these catB cysteine proteases may also be implicated in the degradation of epithelial villin [102]. Otherwise, very little is known about the function of *Giardia* cathepsin cysteine proteases (Box 1).

Box 1 ***Giardia* cathepsin cysteine proteases.**

The term cathepsin was initially used to describe proteases active in a lightly acidic environment. However, as genome sequencing of different species has progressed, it has become evident that not all cathepsin-like proteases are active at an acidic pH. Cathepsin cysteine proteases consist of a catalytic diad of a cysteine and a histidine residue, whereby the histidine residue donates an electron to the cysteine residue to make it a stronger nucleophile (reviewed in [103]). Cathepsin cysteine proteases are divided down into two superfamilies: the cathepsin-L(catL)-like and the cathepsin-B-like superfamilies. Cathepsin B-like cysteine proteases contain a unique ~20-amino-acid insertion referred to as the occluding loop; this structure allows the protease to function as an endo- and exopeptidase. The *Giardia* genome contains genes for numerous catB and catL proteases [104]. Interestingly, the Giardia catB protein appears to lack the occluding loop present in human catB [105]. Prior to an immunomodulatory role for *Giardia* catB proteases, very little was known about *Giardia* cathepsin cysteine proteases. Indeed, it was demonstrated that these factors were upregulated following exposure to *in vitro* intestinal epithelial monolayers and they played a role in parasite encystation and excystation [106]. Ongoing research has demonstrated that *Giardia* cathepsin-like cysteine proteases induce the myosin light chain kinase (MLCK)-mediated breakdown of cytoskeletal villin [102]. Future studies should continue to elucidate the role of *Giardia* cathepsin cysteine proteases in disease and their regulation within the parasite For example, *Toxoplasma gondii* catB proteases actually require catalytic activation via the parasite's catL proteases [107]. It remains to be seen whether similar effects are observed with *Giardia* catB proteases.

**Figure 1:**
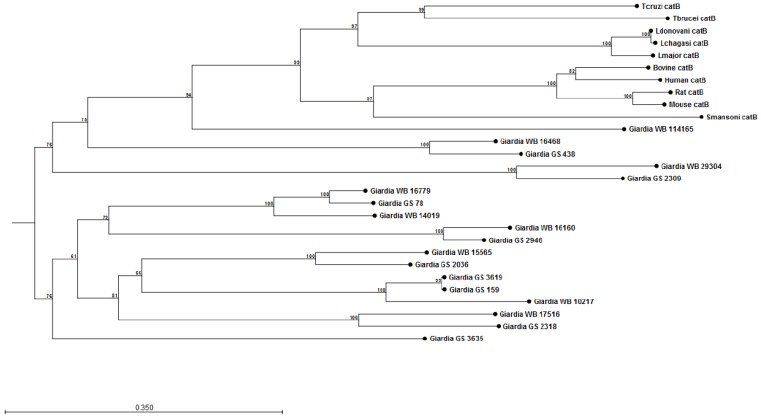
Phylogenetic tree and bootstrap values of *Giardia* WB and *Giardia* GS/M cathepsin B cysteine proteases. Proteases were compared against human, bovine, mouse, rat, *Schisotoma mansonii*, *Leishmania major*, *L. donovani*, *L. chagasi*, *Trypanosoma brucei*, and *T. cruzi* catB cysteine proteases. Alignment and phylogenetic trees of cathepsin B cysteine proteases were assembled using ClustalW and CLC Sequence Viewer (Qiagen). These observations indicate that *Giardia* isolates may contain unique catB cysteine proteases.

**Figure 2:**
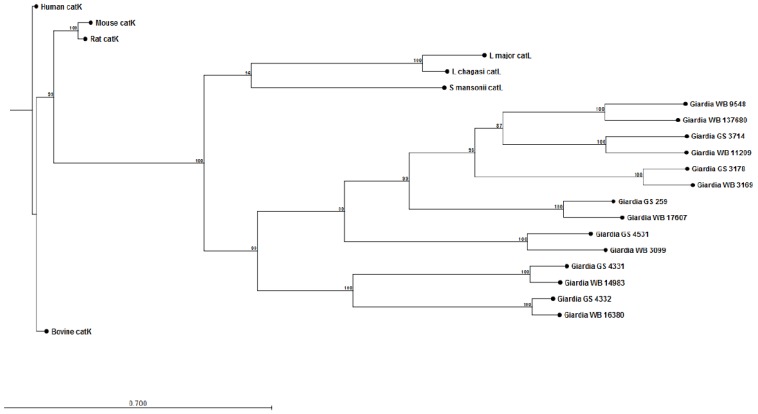
Phylogenetic tree and bootstrap values of *Giardia* WB and *Giardia* GS/M cathepsin L cysteine proteases. Proteases were compared against human, bovine, mouse, rat, *Schisotoma mansonii*, *Leishmania major*, and *L. chagasi* catL cysteine proteases. Alignment and phylogenetic trees of cathepsin L cysteine proteases were assembled using ClustalW and CLC Sequence Viewer (Qiagen).

The construction of preliminary phylogenetic trees using ClustalW [108] for *Giardia* cathepsin B (catB) (Figure 1) and cathepsin L (catL) (Figure 2) cysteine proteases of sequenced parasite isolates used in our above study (see Cotton *et al* [101]) suggests certain parasite isolates may contain unique catB proteases; this may explain differences in the ability of parasites to degrade CXCL8. However, it should be noted that sequencing of *Giardia* genomes is incomplete and, therefore, construction of these phylogenetic trees requires re-analysis following their completion. These data may lend credence to the hypothesis that certain *Giardia* isolates may possess unique immunomodulatory cathepsin cysteine proteases. The utilization of these phylogenetic trees in association with the Cre/loxP system in *Giardia* trophozoites [109] may aid in the identification of immunomodulatory functions of cathepsin cysteine proteases. In addition, it remains to be determined whether *Giardia* catB proteases modulate or degrade other cytokines or chemokines, as indicated by the above observations that *G. duodenalis* trophozoites reduce tissue concentrations of numerous cytokines and chemokines released from inflamed colonic mucosal biopsy tissues [100]. Indeed, other parasites use cathepsin-like cysteine proteases to modulate host immune responses via the alteration of cytokines or chemokines (reviewed in [105,110]). For example, *Entamoeba histolytica* cysteine proteases alter interleukin-18 [111] and the end-target PMN chemokine C5a [112]. As a result, future studies could investigate other pro-inflammatory mediators targeted by *Giardia* cathepsin cysteine proteases.

CXCL8 is primarily secreted basolaterally by IECs to recruit extravasated PMNs to the basolateral membrane of the intestinal epithelium so subsequent signals can, if necessary, promote PMN transepithelial migration [113,114,115]. Therefore, the immunomodulatory capability of *Giardia* cathepsin cysteine proteases implies that these must be delivered to the basolateral surface of the intestinal epithelium. In the studies discussed above, apical-to-basolateral translocation of cysteine proteases occurred when *Giardia* trophozoites and Caco-2 monolayers were co-incubated with *Salmonella enterica* serovar Typhimurium [101]. These results may also suggest that delivery of immunomodulatory cathepsin cysteine proteases can be facilitated by the presence of *S.* Typhimurium. Another study demonstrated that co-incubation of *Giardia* trophozoites, intestinal epithelial monolayers, and macrophage-like IC-21 cells *in vitro* resulted in basolateral attenuation of CXCL8 [116]; this study did not investigate causal mechanisms. As macrophages are known to increase intestinal epithelial permeability [117], more research is needed to assess whether and how the interaction between parasites, IECs, and immune cells may facilitate the apical-to-basolateral migration of immunomodulatory *Giardia* catB proteases.

#### Modulation of Neutrophil Recruitment and Co-Infections

It is well established that individuals with genetic mutations resulting in defective PMN function are highly susceptible to bacterial and fungal infection [118,119,120], and similar events have been observed during certain experimental GI infections. For example, *in vivo* depletion of PMNs increases mortality due to *C. difficile* infection [121,122], while intestinal PMN influx reduces pathogen burdens from the attaching and effacing pathogen *Citrobacter rodentium* and protects against pathogen-induced diarrheal disease [123]. In contrast, other reports indicate that GI inflammatory responses and PMN infiltration may increase susceptibility to GI infection, and it has been postulated that the development of GI inflammatory responses disrupts resident microbiota populations, which in turn aides pathogen colonization [124]. Research has shown that *S.* Typhimurium outcompetes the host’s resident microbiota during intestinal inflammatory responses to facilitate its colonization [125,126,127,128]. Similarly, attenuated PMN recruitment *in vivo* reduces colonization by *Campylobacter jejuni* [129] and *C. rodentium* [130]. In addition, PMN recruitment has been shown to aggravate experimental colitis [131]; this may occur via the PMN's ability to induce protective responses within IECs via the induction of hypoxia-inducible factor (HIF) [132] or the secretion of interleukin-22 (IL-22) [133]. In this context, the above observations that certain *Giardia* infections modulate PMN recruitment require further investigation in the context of GI co-infection. Specifically, experiments need to ascertain whether *Giardia*-mediated modulation of PMN recruitment into intestinal tissues is of benefit or detriment to a host co-infected with another GI pathogen. This may, ultimately, be dependent upon the co-infecting GI pathogen.

### 4.3. Giardia and L-Arginine

L-arginine is utilized for a variety of cellular processes and signaling events; it is incorporated into various proteins and is a precursor substrate for various other molecules [134]. During homeostasis, L-arginine levels are maintained by endogenous production in the intestine and kidneys [134]. Several pathogens have been found to compete with their host for L-arginine during infection, and, therefore, exogenous sources of the amino acid are required in these instances [135]. Indeed, L-arginine is a primary source of energy for *Giardia* trophozoites [136], and restoration of host arginine levels via ornithine supplementation during oral rehydration therapy has been proposed to be beneficial in the treatment of symptomatic giardiasis [137]. Nitric oxide (NO) is also important in prompt parasite eradication in *in vivo G. duodenalis* infections in mice [138,139,140]. Following exposure to intestinal epithelial monolayers, *Giardia* trophozoites quickly upregulate the expression of two enzymes: arginine deiminase and ornithine carbomyl transferase (OCT). These enzymes are important in parasite arginine metabolism and effectively outcompete host enzymes [141,142]. Depletion of L-arginine levels has been shown to affect multiple cellular processes and results in the modulation of host immune responses; this includes decreased production of nitric oxide, modulation of T-cell and dendritic cell function, and cessation of intestinal epithelial proliferation (discussed below).

#### Parasite Arginine Consumption Inhibits NO Production

NO production is initiated by the enzymatic conversion of L-arginine into L-citrulline by three nitric oxide synthase (NOS) isoforms: neuronal NOS (nNOS or NOS1), inducible NOS (iNOS or NOS2), or endothelial NOS (eNOS or NOS3) [143]. In IECs, NO is largely produced by iNOS, and is upregulated following exposure to various host- or pathogen-derived pro-inflammatory stimulatory processes [144,145]. NO has anti-microbial activity against numerous bacterial and parasitic pathogens [146,147,148], and *in vitro* experiments have demonstrated NO and its end-products are cytostatic to *Giardia* trophozoites and inhibit their encystation and excystation [48,141]. Exposure to parasites resulted in the initial upregulation of iNOS mRNA in *in vitro* intestinal epithelial monolayers [149], but human studies suggest that infection may also result in the downregulation of iNOS expression [150]. L-arginine consumption by *Giardia* trophozoites prevents the IEC-mediated production of iNOS-mediated NO production [48,141]. Moreover, assemblage A and B *Giardia* trophozoites produce a flavohemoglobin capable of degrading NO and attenuating T-cell proliferation [149]. Collectively, these results demonstrate that *Giardia* trophozoites possess multiple mechanisms aimed at decreasing their exposure to NO.

It remains to be determined how the consumption of L-arginine and the concomitant loss of NO during *Giardia* infections may contribute to the modulation of host immune responses and/or susceptibility to co-infecting GI pathogens. Indeed, NO has multiple roles in modulating host immune responses and targeting GI pathogens. For example, animals deficient in iNOS are highly susceptible to infection with *Listeria monocytogenes* [151]. Separately, *in vivo* models of colitis have shown that the activation of iNOS and the subsequent NO production contribute to the resolution of inflammation [152] and attenuate pro-inflammatory responses [153]. In contrast, genetic deletion or pharmacological inhibition of iNOS has been shown to protect against dextran sodium sulfate (DSS)-induced colitis [154,155]. These results suggest that the immunomodulatory capacity of iNOS and NO may depend on the model of GI inflammation. Additional experiments are required to determine how the *Giardia*-mediated inhibition of NO production may, potentially, modulate host inflammatory responses and/or susceptibility to GI infection. It has also been demonstrated that NO production during GI infection can induce anion secretion and, therefore, cause diarrheal disease [156]. However, NO has also been shown to inhibit intestinal trafficking of the cystic fibrosis transmembrane conductance regulator (CFTR) protein in intestinal epithelial monolayers and, therefore, may inhibit anion secretion [157]. As numerous GI pathogens induce NO, it is possible that *Giardia* infections protect against the development of diarrheal disease via this mechanism. However, additional studies are required to confirm this hypothesis.

### 4.4. Intestinal Epithelial Cell Death

Changes in intestinal epithelial cellular proliferation are essential responses to GI infection and facilitate the removal of damaged and/or pathogen-infected cells; however, GI pathogens can alter the kinetics of epithelial cell death and turnover to facilitate their colonization and subsequent invasion (reviewed in [158,159,160,161]). For example, enteroinvasive *Escherichia coli*, *Salmonella* sp., and *Shigella* sp. initially suppress and, subsequently, induce various forms of intestinal epithelial cell death to facilitate replication and dissemination within their host, respectively; this can be associated with the activation of pro-survival pathways such as the NF-κB pathway [162,163,164,165,166]. *Giardia* infections can inhibit intestinal epithelial proliferation and, subsequently, induce intestinal epithelial apoptosis; however, the pathophysiological processes may differ from those of other GI pathogens discussed above. L-arginine is involved in cellular proliferation via its conversion into polyamines [167], and *Giardia* arginine deiminase-mediated consumption of arginine has been associated with the inhibition of *in vitro* IEC proliferation [137]; this consumption was proposed to reduce intestinal epithelial cell turnover and create a more stable environment for the parasite [137]. Contrastingly, increases in intestinal epithelial proliferation have been reported in *in vivo G. duodenalis* GS/M mouse infections [168]; therefore, it remains to be determined whether the consumption of arginine by parasites inhibits IEC proliferation. Other reports have demonstrated that *Giardia* trophozoites induce IEC apoptosis via the activation of cysteinyl asparate proteases (caspases) through mechanisms that remain incompletely understood [35,37,39]. However, it remains to be determined how these pathophysiologic processes induced by *Giardia* potentially modulate host immune responses and their interaction during GI co-infections. It is possible that *Giardia*-mediated upregulation of intestinal epithelial cell death may increase the expulsion of co-infecting GI pathogens. It is also currently unknown whether *Giardia* trophozoites modulate pro-inflammatory signaling cascades, such as the NF-κB pathway, in IECs to delay the induction of cell death. Caspase proteins inactivate or degrade various proteins associated with the NF-κB signaling cascade [169,170,171]. Research needs to determine whether *Giardia* may degrade pro-inflammatory transcription factors, thereby preventing bacterial pathogens from initially inhibiting cell death cascades within IECs to allow for their replication prior to dissemination into deeper host tissues.

### 4.5. Dendritic Cells

Dendritic cells (DCs) are essential to the induction of adaptive immune responses and/or tolerance. Following their activation, DCs become immunogenic antigen-presenting cells capable of promoting the expansion and differentiation of naïve T-cells into effector T-cells via a three-step process. DCs consume and process antigen, couple it to major histocompatibility complexes (MHC), and, subsequently, present this to naïve T-cell populations; in addition, DCs also use co-stimulatory molecules, such as CD80 and CD86, and produce mediators, such as cytokines, to influence the differentiation of naïve T cells in various subsets (reviewed in [172,173]). Within the GI tract, especially in the distal small intestine, DCs directly sample luminal contents via the extension of dendrites between adjacent IECs [174,175]. Research to date has produced conflicting results on how *Giardia* trophozoites affect DC activation and their ability to induce and/or modulate effector immune responses. The co-incubation of *Giardia* assemblage B GS/M trophozoite extracts and murine bone marrow-derived DCs *in vitro* resulted in the upregulation of co-stimulatory CD40, and to a lesser extent, CD80 and CD86; moreover, these extracts altered DC responses to toll-like receptor (TLR) ligands, whereby parasites reduced the expression of MHC Class II, CD80, and C86, decreased the secretion of IL-12, and enhanced IL-10 production via activation of the PI3K pathway [176]. In contrast, separate experiments found that the *Giardia* homolog of immunoglobulin protein (BiP) triggered the expression of MHC Class II molecules and concomitantly resulted in the secretion of TNFα, IL-12, and IL-6 via several pro-inflammatory signaling cascades in *in vitro* murine dendritic cells [177]. Experiments using assemblage A *Giardia* WB trophozoites demonstrated that the parasite decreases the production of IL-12p40, IL-12p70, and IL-23 by human DC *in vitro* and the expression of co-stimulatory molecules and human leukocyte antigen (HLA) DR (HLA-DR) while enhancing the production of anti-inflammatory IL-10; interestingly, DCs incubated with these parasites and concurrently exposed to TLR2 ligands enhanced IL-12p40, IL-23, and IL-10 production [178]. Separately, arginine depletion induced by the same parasite isolate was shown to reduce the surface expression of CD83 and CD86, decrease the secretion of IL-10 and IL-12p40, and enhance TNFα production in *in vitro* human DCs [179]. In other experiments, the *in vitro* co-incubation of bovine DCs and a mixture of *Giardia* assemblage A and E trophozoites resulted in elevated MHC Class II molecules, TGF-β, TNFα, IL-10, and IL-4; these DCs are able to induce T-cell proliferation [180]. Collectively, these results demonstrate that *Giardia* trophozoites are capable of modulating DC cell function. Future studies should compare and contrast DC function and activation following exposure to different *Giardia* isolates and assemblages.

### 4.6. Macrophages

Ongoing research has demonstrated these macrophages change their function based on endogenous and exogenous stimuli within the local tissue environment; this also results in the altered expression of several surface markers and leads to their classification into subgroups, commonly known as M1 and M2 macrophages [181,182]. M1 macrophages have been labelled as “inflammatory macrophages” that produce various inflammatory mediators and molecules, such as TNFα, whereas the M2 macrophage phenotype is often thought to antagonize host pro-inflammatory responses, including the production of nitric oxide, and can result in the expression of Arginase-1 [183]. However, current research suggests these cell types do not exist as distinct entities, but rather as a continuum of differing phenotypes [184]. To date, very little research has examined how *Giardia* infections modulate macrophage phenotypes during infection, and only one study has shown that *in vivo Giardia* assemblage B infections result in the accumulation of macrophages positive for both NOS2 and Arginase-1 [185]. Additional research is required in order to investigate how *Giardia* infections induce this unique macrophage phenotype, and, moreover, whether these macrophages are observed during human *Giardia* infections or *in vivo* assemblage A or *Giardia muris* infections. Future studies are also required to assess whether a *Giardia-*induced switch to macrophage phenotype, if present, may alter susceptibility to GI co-infection. As individuals with mutations in cytokines associated with M1 macrophage polarization are more susceptible to infection by numerous microorganisms [186,187], *Giardia*-induced changes to macrophage phenotypes may significantly affect susceptibility to a variety of infections. For example, the intracellular replication of *S.* Typhimurium is greatly impaired in monocyte-derived macrophages with an M1 phenotype [188]. Moreover, macrophage Arginase-1 expression has been found to limit helper Th2-mediated immune responses and fibrosis during *in vivo Schistosoma mansonii* infection [189]. Collectively, these studies highlight the need for additional research examining the interaction between *Giardia* and host macrophages.

## 5. *Giardia* and Distant Site Co-Infections

Although this review is focused on the immunomodulatory effects of *Giardia* in GI tissues and its potential effects on host responses during GI co-infection, it is important to also consider whether this parasite may alter host immune responses to microbial pathogens at distant organ sites. Such effects have been observed with soil-transmitted helminth infections modulating host immune responses to various pathogens, including *Plasmodium* sp., *Mycobacterium tuberculosis*, and human immunodeficiency virus (HIV) (reviewed in [190]). It is possible that the immunomodulatory mechanisms of *Giardia* may also modify host responses to other pathogens. Human studies have demonstrated that *Giardia* infections reduce serum levels of serum CRP [50], thereby indicating that these infections are capable of modulating systemic immune responses. However, studies have not examined whether *Giardia* infections modulate systemic immune responses to microbial pathogens at distant tissue sites. Currently, a few studies have demonstrated that *Giardia* infections can be observed in association with blood-borne parasites, such as *Plasmodium* sp. [191] and *Leishmania* sp. [192]. In addition, individuals with immune deficiencies, such as HIV/AIDS, may be more susceptible to *Giardia* infection [193,194,195]. However, our understanding of how immunomodulatory mechanisms employed by *Giardia* may alter infection dynamics is poor, and research is this field is sorely needed.

## 6. Summary

**Figure 3:**
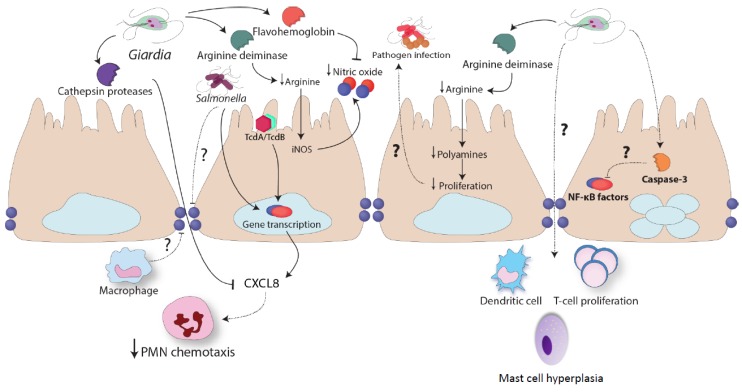
Immunomodulation by *Giardia* sp. *Giardia* infections have been shown to attenuate granulocyte infiltration *in vivo* following intra-rectal instillation of *Clostridium difficile* toxin A and B (TcdA/TcdB). *Giardia* trophozoites release cathepsin cysteine proteases that attenuate PMN chemotaxis; it remains unknown how these proteases cross the intestinal epithelial barrier. Arginine deiminase (ADI) released by the parasite consumes L-arginine and this results in attenuated nitric oxide (NO) production. Furthermore, flavohemoglobins released by the parasite decrease the levels of NO. *Giardia* arginine deiminase also decreases intestinal epithelial proliferation, and this may affect the ability of other pathogens to colonize the intestinal tract. Similarly, during trophozoite-induced intestinal epithelial apoptosis, the activation of caspase proteins may cleave pro-inflammatory transcription factors. Multiple reports have shown that *Giardia* trophozoites modulate dendritic cell and helper T cell function, and cause mast cell hyperplasia. Additional research is required to characterize the mechanisms and consequences of these observations.

Our understanding of *Giardia* infections is continually improving, and it is now apparent that parasites possess multiple mechanisms capable of modulating host intestinal inflammatory responses, yet much remains to be uncovered (Figure 3). Moreover, human studies indicate that infections can be commonly found in association with other pro-inflammatory GI pathogens, especially in developing countries, and, in certain instances, infections have been shown to protect against the development of diarrheal disease. It remains to be causally demonstrated whether the parasites' immunomodulatory mechanisms are responsible for the attenuating diarrheal disease within their host. Therefore, future studies need to assess the immunomodulatory mechanisms of *Giardia* infections in the context of the attenuation of diarrheal disease. Interestingly, experimental evidence to date suggests that certain immunomodulatory capabilities of the parasite are associated with different *Giardia* assemblages, and future research needs to assess whether similar effects are observed in humans. These observations may lend further support to the discussion on Giardia assemblage A and B speciation. Finally, it remains to be seen whether *Giardia* infections are capable of modulating systemic immune responses or immune responses at distant organ tissues. As the highest incidence rates of *Giardia* infections geographically overlap with various other microbial pathogens, future research needs to consider whether *Giardia* infections modulate host immune responses to pathogens at sites distant from the GI tract.
